# Animal control by spiking neural networks evolved with a genetic algorithm

**DOI:** 10.1186/1471-2202-16-S1-P237

**Published:** 2015-12-18

**Authors:** Borys Wróbel, Ahmed Abdelmotaleb, Neil Davey, Volker Steuber

**Affiliations:** 1 Evolutionary Systems Group, Adam Mickiewicz University, Poznan, Poland; 2 Systems Modeling Group, IOPAN, Sopot, Poland; 3 Biocomputation Research Group, University of Hertfordshire, Hatfield, UK

## 

The ability to search for resources is an example of a minimally cognitive behavior---a behavior shown by even the simplest animals, and that can be explored using simple robots [1]. Even very simple networks (such as natural and artificial genetic or neural networks) allow for control of the simplest search behaviors [2]. Moreover, this cognitive task can be made more difficult [3], so it can be seen as a possible stepping step toward advanced cognitive skills, both in biology and robotics [1].

In biology, the topology and synaptic weights of simple networks is rather evolved than learned. Here we used an artificial life platform called GReaNs that allows to use a genetic algorithm to evolve simple spiking neural networks (SNNs) using a mixed bio-inspired paradigm - the way the topology and weighs are encoded in artificial genomes is inspired by genetic networks, but computational units in the network are modeled as either leaky integrate and fire neurons with a fixed threshold or adaptive-exponential integrate and fire neurons [3].

We evolved SNNs with GReaNs to control simple artificial robots (animats) whose task was to search for targets in a 2-dimensional artificial environment. The targets can be seen as food pellets from which food diffuses, and is sensed by robot's two smell/taste sensors, on the left and right front. The robot also has two actuators which generate thrust on the left and right back; when the thrust on one side is larger, the animat moves in a circle; when the thrusts are the same, the robot goes forward in a straight line.

We have designed three ways to present the strength of the sensed signal to the network, and three ways to relate the thrust generated by the two actuators to the activity of the corresponding two motor neurons in the network. All the tested setups allowed us to evolve robots with correct search behavior. Out of three setups for sensors, two can be seen as biologically realistic. In one of them, easier to evolve, the sensory information was preprocessed. Pre-processing consisted of calculating the difference and the sum of the smell sensed by the sensors, using the results as arguments of two sigmoid functions to obtain two values that determined the percentages of activation of two populations of 100 primary sensory neurons, each connected with one synapse (with the same weight) to secondary sensory neurons. In the second setup, a Hill function was used to map the smell of two sensors as current injection to two sensory neurons (in other words, here there was no pre-processing of the difference between the smell strength on two sides of the robot). Out of two setups for actuators we tested, again two were biologically realistic. It proved easier to evolve a setup in which constant thrust was generated in an actuator when the corresponding motor neurons spiked. In the less evolvable approach, the thrust was determined by summing the number of spikes of the corresponding motor neuron over a sliding temporal window.
